# FN-OCT: Disease Detection Algorithm for Retinal Optical Coherence Tomography Based on a Fusion Network

**DOI:** 10.3389/fninf.2022.876927

**Published:** 2022-06-16

**Authors:** Zhuang Ai, Xuan Huang, Jing Feng, Hui Wang, Yong Tao, Fanxin Zeng, Yaping Lu

**Affiliations:** ^1^Department of Research and Development, Sinopharm Genomics Technology Co., Ltd., Jiangsu, China; ^2^Department of Ophthalmology, Beijing Chao-Yang Hospital, Capital Medical University, Beijing, China; ^3^Medical Research Center, Beijing Chao-Yang Hospital, Capital Medical University, Beijing, China; ^4^Department of Clinical Research Center, Dazhou Central Hospital, Sichuan, China

**Keywords:** fusion network, optical coherence tomography, attention mechanism, retinal disease, model interpretability

## Abstract

Optical coherence tomography (OCT) is a new type of tomography that has experienced rapid development and potential in recent years. It is playing an increasingly important role in retinopathy diagnoses. At present, due to the uneven distributions of medical resources in various regions, the uneven proficiency levels of doctors in grassroots and remote areas, and the development needs of rare disease diagnosis and precision medicine, artificial intelligence technology based on deep learning can provide fast, accurate, and effective solutions for the recognition and diagnosis of retinal OCT images. To prevent vision damage and blindness caused by the delayed discovery of retinopathy, a fusion network (FN)-based retinal OCT classification algorithm (FN-OCT) is proposed in this paper to improve upon the adaptability and accuracy of traditional classification algorithms. The InceptionV3, Inception-ResNet, and Xception deep learning algorithms are used as base classifiers, a convolutional block attention mechanism (CBAM) is added after each base classifier, and three different fusion strategies are used to merge the prediction results of the base classifiers to output the final prediction results (choroidal neovascularization (CNV), diabetic macular oedema (DME), drusen, normal). The results show that in a classification problem involving the UCSD common retinal OCT dataset (108,312 OCT images from 4,686 patients), compared with that of the InceptionV3 network model, the prediction accuracy of FN-OCT is improved by 5.3% (accuracy = 98.7%, area under the curve (AUC) = 99.1%). The predictive accuracy and AUC achieved on an external dataset for the classification of retinal OCT diseases are 92 and 94.5%, respectively, and gradient-weighted class activation mapping (Grad-CAM) is used as a visualization tool to verify the effectiveness of the proposed FNs. This finding indicates that the developed fusion algorithm can significantly improve the performance of classifiers while providing a powerful tool and theoretical support for assisting with the diagnosis of retinal OCT.

## 1. Introduction

Both age-related macular degeneration (AMD) and diabetic macular oedema (DME) are highly common retinal diseases that cause blindness. AMD is the result of the inactivation and degeneration of macular photoreceptor cells and is one of the major causes of irreversible vision loss. Drusen is an early manifestation of AMD, and without timely diagnosis and early intervention, it can lead to the progression of the disease to its middle and late stages. Therefore, early drusen detection and treatment can delay or stop the transition to advanced AMD. In advanced wet AMD, the most common form of blindness is choroidal neovascularization (CNV).

Optical coherence tomography (OCT) has been applied in clinical ophthalmology since the 1990s (Hee et al., 1995) and has enabled the attainment of images similar to those of *in vivo* eye histopathology. OCT is also a high-resolution, noninvasive biological tissue imaging technology. With the rapid development of this technology, our ability to identify ophthalmic diseases has also gradually improved (Schmitt, 1999). During the actual clinical diagnosis process, it is necessary for professional doctors to conduct imaging analyses on retinal OCT images to make accurate judgments. However, differences in the levels of expertise among doctors in different countries and regions can lead to faulty diagnoses. For most eye diseases that lead to blindness, early diagnosis and treatment can prevent them from progressing to the degree of visual impairment. Therefore, we need to use medical image recognition machines to help identify such diseases.

Compared with single-algorithm models, the advantage of an ensemble learning model is that it can organically integrate multiple single-algorithm models to obtain a unified and integrated algorithm model to obtain more accurate, stable, and strong results. Early in the field of machine learning, most major competitions used ensemble learning to obtain higher evaluation indicators (Illy et al., 2019; Lian et al., 2020; Rajadurai and Gandhi, 2020). Generally, the combination strategies of ensemble learning algorithms based on machine learning include voting mechanisms (Gao et al., 2021) and arithmetic weighted averages (Sun et al., 2015). These fusion methods fuse and output results through simple linear combinations. For complex medical image samples encountered in real life, a simple linear combination is difficult to adapt.

Therefore, the following difficulties exist when constructing an algorithm model for retinal OCT disease detection based on a fusion network (FN).

1. How to address complex medical scene image data in a linear combination strategy.
2. How to put forward a nonlinear combination strategy for medical scene image data.

To solve the above difficulties, this paper proposes two linear fusion strategies and a nonlinear fusion strategy.

1. The F1 value obtained by each base classifier on the validation set is used as a parameter to set its weight.
2. It is proposed to use multiple trainable weight parameters to automatically obtain solutions according to the utilized loss function, which can adapt to different complex scenarios.
3. The weights of different base classifiers are calculated by using a nonlinear function involving deep learning.

## 2. Related Work

In recent years, numerous algorithms have been used to detect retinal OCT lesions (Apostolopoulos et al., 2017; Karri et al., 2017; Yoo et al., 2019; Das et al., 2020), and these diagnostic methods can be roughly divided into two categories.

The first category contains algorithmic retinal OCT lesion detection methods based on machine learning. This type of approach employs commonly used image processing algorithms (multiscale histograms of oriented gradients, scale-invariant feature transformations, local binary patterns (LBPs), etc.) to extract image features (Liu et al., 2011; Albarrak et al., 2013; Srinivasan et al., 2014; Lemaître et al., 2015; Sankar et al., 2016). Then, the extracted image features are input into commonly used machine learning algorithms (support vector machines (SVMs), random forests, etc.), and these algorithms determine the category of the image. Alsaih (Alsaih et al., 2016) extracted the directional gradient histogram and local binary mode of OCT and combined them into a set of different feature vectors, which were input into a linear SVM classifier to predict image categories. Sun et al. (2017) proposed a universal method for automatically aligning and clipping retinal regions; then, the global representation of the given image was obtained by using sparse coding and a spatial pyramid. Finally, a multiclass linear SVM classifier was used to classify dry AMD and DME.

However, the commonly used image feature extraction algorithms tend to lose large amounts of image information and thus cannot fully represent image features. In addition, images based on retinal OCT lesions exhibit certain similarities, resulting in poor performance for the commonly used image processing algorithms. Thus, the second category includes retinal OCT lesion detection methods based on deep learning (Karri et al., 2017; Lee et al., 2017; Rasti et al., 2018; Treder et al., 2018; Fang et al., 2019; Huang et al., 2019; Hassan et al., 2021; He et al., 2021). Rong et al. (2019) first removed noise from an original image, then generated image masks by using thresholding and morphological dilation and then used the noise-free image and image masks to generate an alternative image. Finally, the alternative image was input into a convolutional neural network (CNN) to predict the category of the original input image. Fang et al. (2019) first input an original image into a lesion detection network to generate a lesion attentional map and then incorporated this map into a classification network to enhance the contribution capacity of local convolution. Weighted by the lesion attention map, the classification network could further accelerate the network training process and improve its OCT classification ability by utilizing information from local lesion-related regions. Kermany et al. (2018) used an InceptionV3 network pretrained on ImageNet to classify OCT images. Das et al. (2020) developed a classifier based on a semi-supervised generative assumption network; this approach can be used for automatic diagnosis with limited marker data. The framework consists of a generator and a discriminator. Learning between these components helps build a generalized classifier to predict retinal disease categories.

The advantages and disadvantages of the above two methods are summarized in Table 1.

**Table 1:** Advantages and disadvantages of retinal OCT methods utilizing deep learning and machine learning.

**Method**	**Advantages**	**Disadvantages**
Machine learning	Works better on small data, less financially and computationally expensive, easier to explain.	Low accuracy, require complex feature engineering.
Deep learning	High precision, no need for feature engineering, strong adaptability, easy to migrate.	Require a large number of training datasets, high-end GPUs; “black box” models.

Therefore, the construction of a retinal OCT disease detection algorithm based on an FN offers several contributions, as follows.

1. Three fusion solutions that can be used in the processes of multimodal fusion and multinetwork fusion are proposed.
2. Fully automatic retinal OCT disease image detection is achieved without manual intervention.
3. The accuracy is increased by 5.3%, and the accuracy and area under the curve (AUC) reached 98.7 and 99.1%, respectively.
4. Common network models are used in conjunction with attention mechanisms in retinal OCT scenarios.
5. The gradient-weighted class activation mapping (Grad-CAM) algorithm is used to verify the validity of the fusion network.

## 3. Disease Detection Algorithm for Retinal OCT Based on an FN

### 3.1. System Architecture

**Figure 1:**
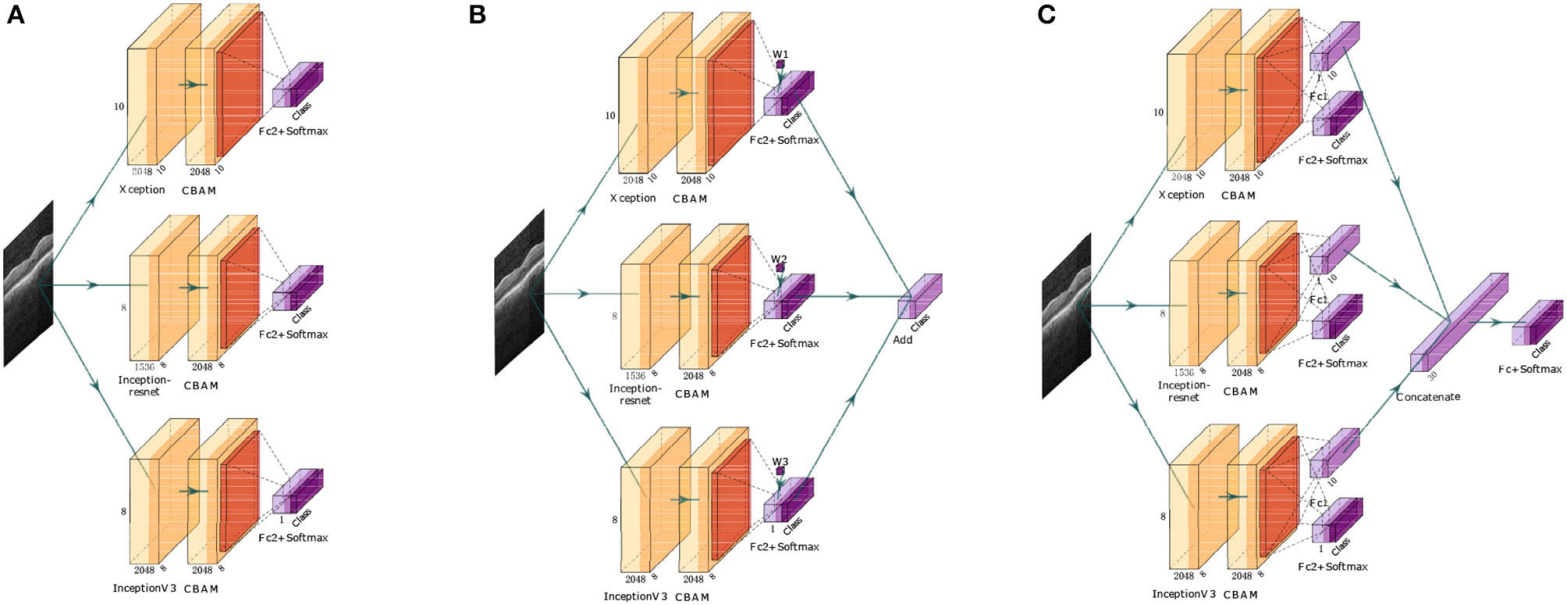
FN. ‘Class’ is the number of categories required by the algorithm, ‘CBAM’ is the attention mechanism, ‘Fc + Softmax’ is the fully connected output layer, “Add” is an addition operation, and ‘Concatenate’ is the splicing operation. **(A)** Is the first fusion (FN-F1-OCT), **(B)** is the second fusion (FN-Weight-OCT), and **(C)** is the third fusion (FN-Auto-OCT). The network architecture is drawn by using ‘PlotNeuralNet’ (https://github.com/HarisIqbal88/PlotNeuralNet).

The proposed retinal OCT disease detection algorithm based on an FN adopts three methods during model fusion, as shown in Figures 1A–C. In the first method, FN-F1-OCT outputs the prediction results of the base classifiers (Xception, Inception-ResNet, and InceptionV3) and sets the weight values through the F1 values of these three base classifiers. In the second method, the FN-Weight-OCT model sets three trainable variables for the three base classifiers during training. As the network model is trained, the weight parameters change accordingly. In the third method, during the training process of the FN-Auto-OCT model, the three base classifiers connect two fully connected layers. One fully connected layer is used to directly predict the results, the output results of the other fully connected layer are spliced, and the final prediction of the algorithm is automatically determined based on the spliced summary result. FN-Weight-OCT and FN-Auto-OCT exhibit some differences in the weight parameter calculation processes. FN-Weight-OCT has a corresponding weight parameter “w” for each base classifier, and the weight parameter “w” is iteratively updated by the loss function. FN-Auto-OCT first splices the output results of each base classifier and then connects the spliced results to a final output layer; the weight values of the base classifiers are embedded in the calculation of the fully connected layer, which is a fully automated underlying calculation method.

The first implementation of the FN algorithm (FN-F1-OCT) uses the F1 value obtained by each base classifier on the validation set to calculate the weight values. The weight value calculation method for the Xception, Inception-ResNet, and InceptionV3 base classifiers is shown in Formula 1.

(1) $$\begin{array}{l} Wi=\frac{F1\text{\_}list\left[i\right]}{\left(\sum_{p=1}^{q}F1\text{\_}list\left[p\right]\right)}+\left(F1\text{\_}list\left[i\right]-\frac{\sum_{p=1}^{q}F1\text{\_}list\left[p\right]}{q}\right)*n \end{array}$$

where F1_list[i] represents the F1 value obtained by each base classifier on the verification set and $\frac{F1\text{\_}list\left[i\right]}{\left(\sum_{p=1}^{q}F1\text{\_}list\left[p\right]\right)}$ represents the ratio of the F1 values of each base classifier to the sum of the F1 values of the three base classifiers. $\frac{\sum_{p=1}^{q}F1\text{\_}list\left[p\right]}{q}$ represents the average F1 value of each base classifier. $\left(F1\text{\_}list\left[i\right]-\frac{\sum_{p=1}^{q}F1\text{\_}list\left[p\right]}{q}\right)*n$ represents the difference between the F1 value of each base classifier and the average of the F1 values of the three base classifiers, and n is a hyperparameter that expands the differences among the base classifiers. It is assumed that the predicted probabilities of the three base classifiers for the different categories in each sample are Xception_predict, Inception_ResNet_predict, and InceptionV3_predict; therefore, the calculation method for obtaining the final probability value predicted by the model is shown in Formula 2.

(2) $$\begin{array}{l} \begin{array}{l} Y\text{\_}predict=W1*Xception\text{\_}predict\\ \;+W2*Inception\text{\_}ResNet\text{\_}predict\\ \;+W3*InceptionV3\text{\_}predict \end{array} \end{array}$$

(3) $$\begin{array}{l} \begin{array}{l} Y\text{\_}predict=\frac{X}{\left(X+Y+Z\right)}*Xception\text{\_}predict\\ \;+\frac{Y}{\left(X+Y+Z\right)}*Inception\text{\_}ResNet\text{\_}predict\\ \;+\frac{Z}{\left(X+Y+Z\right)}*InceptionV3\text{\_}predict \end{array} \end{array}$$

The second implementation of the FN algorithm (FN-Weight-OCT) first defines three variables X, Y, and Z in the network model, corresponding to the Xception, Inception-ResNet, and InceptionV3 base classifiers, respectively, to prevent the algorithm model from predicting that the probability sum of each category is not 1. In this paper, the three variables are processed to obtain the weight values of each base classifier. In the FN, the weight value of the Xception base classifier is $\frac{X}{\left(X+Y+Z\right)}$, and this value corresponds to the W1 parameter in Figure 1B. Similarly, the weight values of the Inception-ResNet and InceptionV3 base classifiers are $\frac{Y}{\left(X+Y+Z\right)}$ and $\frac{Z}{\left(X+Y+Z\right)}$, respectively, and their weight values correspond to the W2 and W3 parameters in Figure 1B. It is assumed that the predicted probabilities of the three base classifiers for the different categories in each sample are Xception_predict, Inception_ResNet_predict, and InceptionV3_predict, so the method of calculating the sample prediction probability values is shown in Formula 3.

The method for calculating the cross-entropy loss value in the network model consists of the following steps.

1. Assume that the output probability of a sample in the Xception base classifier is [m1,m2,m3,m4], where m1+m2+m3+m4 = 1.
2. Assume that the output probability of a sample in the Inception-ResNet base classifier is [n1,n2,n3,n4], where n1 ++ n2 + n3 + n4 = 1.
3. Assume that the output probability of a sample in the InceptionV3 base classifier is [p1, p2, p3,p4], where p1 + p2 + p3 + p4 = 1.
4. Assume that the real label of the sample is [1,0,0,0] and that the loss value is a cross-entropy loss function, so the method of calculating the loss value of the model is shown in Formula 4.

(4) $$\begin{array}{l} Loss=-\ln\frac{\left(X*m1+Y*n1+Z*p1\right)}{\left(X+Y+Z\right)} \end{array}$$

The parameter update method for the three variables (X, Y, and Z) defined in the network model is as follows.

1. The method of calculating the derivative of the loss with respect to X is shown in Formula 5.
  (5) $$\begin{array}{l} \frac{\partial loss}{\partial X}=\frac{Y\left(n1-m1\right)+Z\left(p1-m1\right)}{\left(X+Y+Z\right)\left(X*m1+Y*N1+Z*P1\right)} \end{array}$$
  The update for X is $X\text{\_}new=X-\eta \frac{\partial loss}{\partial X}$. Thus, η is the learning rate.
2. The method of calculating the derivative of the loss with respect to Y is shown in Formula 6.
  (6) $$\begin{array}{l} \frac{\partial loss}{\partial Y}=\frac{X\left(m1-n1\right)+Z\left(p1-n1\right)}{\left(X+Y+Z\right)\left(X*m1+Y*N1+Z*P1\right)} \end{array}$$
  The update for Y is $Y\text{\_}new=Y-\eta \frac{\partial loss}{\partial Y}$. Thus, η is the learning rate.
3. The method of calculating the derivative of the loss with respect to Z is shown in Formula 7.
  (7) $$\begin{array}{l} \frac{\partial loss}{\partial Z}=\frac{X\left(m1-p1\right)+Y\left(n1-p1\right)}{\left(X+Y+Z\right)\left(X*m1+Y*N1+Z*P1\right)} \end{array}$$
  The update for Z is $Z\text{\_}new=Z-\eta \frac{\partial loss}{\partial Z}$. Thus, η is the learning rate.

The third implementation of the FN algorithm (FN-Auto-OCT) has four output parts, corresponding to the outputs of Xception, Inception-ResNet, InceptionV3, and the FN. In this paper, only the output of the FN is used as the final output result of the algorithm. The outputs of the other three parts are used to backpropagate the three base classifiers to prevent the gradient update process from becoming too slow.

The cross-entropy loss function values of the four output parts in the network model are calculated as follows.

1. The calculation of the cross-entropy loss function for the Xception base classifier is shown in Formula 8.
  (8) $$\begin{array}{l} \begin{array}{l} Loss1=categorical\text{\_}crossentropy(\\ \;Xception\text{\_}output\text{\_}value,reallabel) \end{array} \end{array}$$
2. The calculation of the cross-entropy loss function for the Inception-ResNet base classifier is shown in Formula 9.
  (9) $$\begin{array}{l} \begin{array}{l} Loss2=categorical\text{\_}crossentropy(\\ \;Inception\text{\_}ResNet\text{\_}output\text{\_}value,reallabel) \end{array} \end{array}$$
3. The calculation of the cross-entropy loss function for the InceptionV3 base classifier is shown in Formula 10.
  (10) $$\begin{array}{l} \begin{array}{l} Loss3=categorical\text{\_}crossentropy(\\ \;InceptionV3\text{\_}output\text{\_}value,reallabel) \end{array} \end{array}$$
4. The calculation of the cross-entropy loss function for the fusion model is shown in Formula 11.
  (11) $$\begin{array}{l} \begin{array}{l} Loss4=categorical\text{\_}crossentropy(\\ \;fusion\text{\_}model\text{\_}output\text{\_}value,reallabel) \end{array} \end{array}$$

To calculate the cross-entropy loss function value of the entire network, *Loss* = *Loss*1 + *Loss*2 + *Loss*3 + *Loss*4. This loss value is used as the loss value of the full network.

### 3.2. Model Building and Prediction Module

#### 3.2.1. Data Preprocessing

In this paper, the image preprocessing approach for retinal OCT images includes scaling each image down to 299*299 (*via* nearest-neighbor interpolation), and then each image pixel is normalized according to Formula 12. In this way, the network can easily calculate the image.

(12) $$\begin{array}{l} X_{norm}=\frac{X-X_{min}}{X_{max}-X_{min}} \end{array}$$

#### 3.2.2. Introduction of Various Network Models

We use three highly effective and widely used architectures trained on the ImageNet Large-Scale Visual Recognition Challenge (ILSVRC), InceptionV3, Inception-ResNetV2, and Xception as base classifiers for the FNs (Byeon et al., 2020; Ali et al., 2021; Wang et al., 2021a,b; Yildirim and Çinar, 2021). In theory, these three deep networks can be replaced with other networks based on specific classification tasks.

The InceptionV3 network is a very deep convolutional network developed by Google. In December 2015, InceptionV3 was proposed in the paper "Rethinking the Inception Architecture for Computer Vision" (Szegedy et al., 2016). InceptionV3 reduces the top-5 error rate of ImageNet to 3.5% on the basis of InceptionV2. Compared with InceptionV2, V3 uses n*1 and 1*n convolution cascades to replace the n*n convolution, effectively reducing the number of parameters. Since the introduction of InceptionV3, a large number of researchers have applied this network framework in various fields to help solve problems (Dif et al., 2021; Mahmood and Mahmood, 2021; Rahmanian and Shayegan, 2021; Tembhurne et al., 2021). In agriculture, Zaki et al. (2021) used this algorithm to detect onion disease (purple spots). In medicine, Mijwil (2021) used three architectures (InceptionV3, ResNet, and VGG19) to detect skin cancer images and achieved very acceptable results. After all testing was completed, the best architecture was determined to be InceptionV3. For satellite images, Li and Momen (2021) compared the predictive abilities of four state-of-the-art CNN models, InceptionV3, ResNet50, VGG16, and VGG19, with regard to five different weather events. Overall, InceptionV3 was the best model, with an average accuracy of 92% in detecting such weather systems.

The Inception-ResNet network is a convolution network developed by Google that introduces the idea of ResNet on the basis of inception. In 2016, the network was proposed in "inception-v4, inception RESNET and the impact of residual connections on Learning" (Szegedy et al., 2017); it mainly adds shallow features to high-level features through another branch to achieve the purpose of feature reuse and prevent the gradient disappearance problem encountered by deep networks. Since the introduction of Inception-ResNet, a large number of researchers have applied this network framework in various fields to help solve problems (Al-Antari et al., 2020; Peng et al., 2020; Hung and Su, 2021). In the data preprocessing stage, Bhardwaj et al. (2021) used histogram equalization, optical disc localization, and quadrant cropping for data enhancement. Then, the images of each quadrant were input into Inception-ResNet. Finally, the data of the four quadrants were summarized to obtain the prediction results of the model.

The Xception network is another improvement made by Google after the introduction of Inception. In 2018, Xception was proposed in the paper “xception: deep learning with discrete separable revolutions” (Chollet, 2017). The main innovation is that this network uses a depthwise separable convolution to replace the original convolution operation. Since the introduction of Xception, a large number of researchers have applied this network framework in various fields to help solve problems (Chahal et al., 2021; Chen et al., 2021a; Gurita and Mocanu, 2021). To find the best model that could provide better diagnostic rates for COVID-19, Farag et al. (2021) used random search optimization to tune the hyperparameters of Xception to provide more accurate results than those produced by other techniques. Xu et al. (2021) first transferred weight parameters trained on the ImageNet dataset to the Xception model. A global average pooling layer was then used to replace the fully connected layer of the Xception model. Finally, the extreme gradient boosting (XGBoost) classifier was added to the top layer of the model to output the results.

#### 3.2.3. Model Building

**Figure 2:**
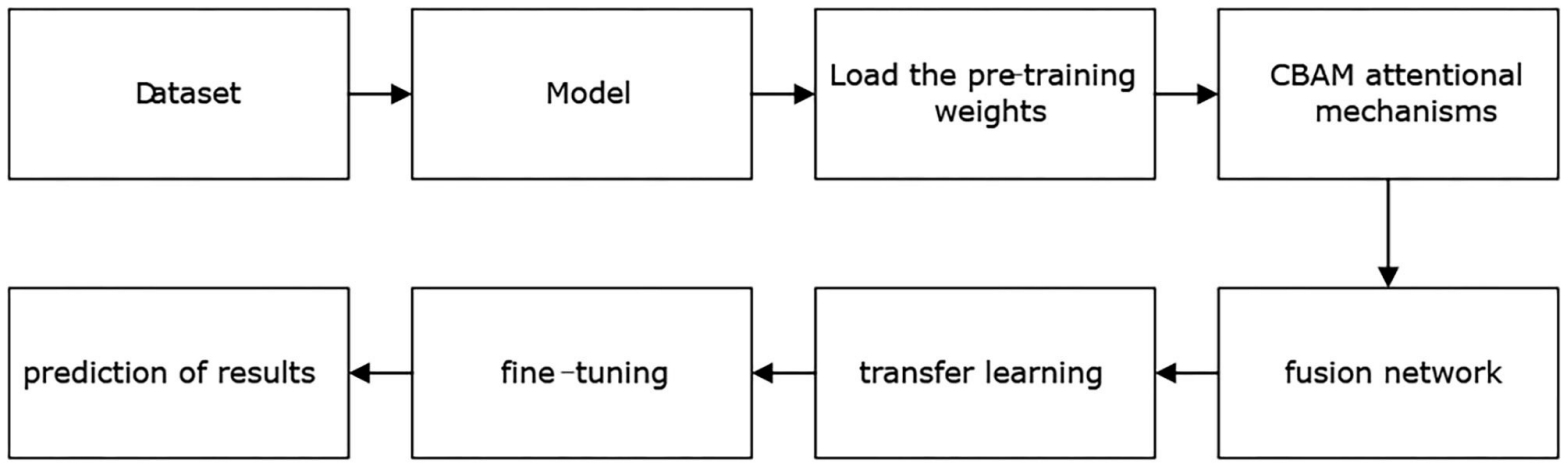
Model building process.

The model training stage is shown in Figure 2, where the “model” part contains three base classifiers corresponding to three FN algorithms. After this, pretraining weight values are loaded based on ImageNet to facilitate network training. The "FN" part contains the different fusion methods of three deep learning algorithms. Next, the algorithm model is transferred to conduct learning, and the process of fine-tuning the mechanism is carried out. Finally, the grade prediction of the algorithm for the given retinal OCT image is output.

Three implementations of the FN in Figure 2 are utilized, including two linear fusion strategies (FN-F1-OCT, FN-Weight-OCT) and one nonlinear fusion strategy (FN-Auto-OCT). These three fusion methods belong to the category of multimodal fusion and are very easy to implement. Multimodal fusion can be divided into early fusion (feature fusion) (Snoek et al., 2005; Pitsikalis et al., 2006; Mou et al., 2021), late fusion (decision fusion, similar to ensemble learning) (Guironnet et al., 2005; Singh et al., 2006), and hybrid fusion. Early fusion fuses the obtained features immediately after they are extracted, and late fusion is performed after each mode outputs its results (such as classification or regression results). Hybrid fusion combines the early fusion and late fusion methods. The linear fusion approach proposed in this paper is a late fusion strategy; that is, after each mode obtains its prediction result, the output results of each mode are fused. Nonlinear fusion is an early fusion method; that is, feature fusion is carried out before each mode outputs its final result.

The most common problem involved in conducting medical image recognition and analysis based on deep learning is the lack of a large labeled medical image dataset. However, transfer learning can solve this problem by applying trained network model weights to medical image analysis through a large dataset (ImageNet). Although medical datasets are different from nonmedical datasets, the low-level features of the images in most image analysis tasks are universal (Sharma and Mehra, 2020), so the weight parameters obtained from large datasets can greatly reduce the cost of data training. Two types of learning are available: transfer learning and fine-tuning.

In transfer learning, all convolution layer parameters of the CNN model trained on a large dataset (for example, ImageNet) are frozen, while the fully connected layer is removed. The convolution layer is used to extract low-level features from the input image. The extracted features are then fed to a classifier to adapt to different application scenarios. During the training process, only the classifier of the model is trained, and all convolution layers are not involved in the training procedure.

Compared with transfer learning, fine-tuning takes the weight parameters of the convolutional layer of a well-trained CNN model as the initial weight parameters and randomly initializes the weight parameters of the classifier at the same time. During this period, the weight parameters of the whole network participate in the training process. The fine-tuning process in this paper is a parameter update procedure based on the weight parameters of the network model obtained by transfer learning.

#### 3.2.4. Attention Mechanism

The convolutional block attention module (CBAM) (Woo et al., 2018) was proposed as a simple and efficient attention module. Given an intermediate feature graph of the utilized network model, attention weight values are successively calculated along the spatial and channel directions and then multiplied by the original input feature graph to adaptively adjust the features. Because the CBAM is a lightweight general-purpose module that can be seamlessly connected to any network feature graph, its parameters are almost negligible. When the CBAM is connected to different network models on t different classification and detection datasets, the final prediction abilities of the models are improved to a certain extent, and their adaptability is strong (Canayaz, 2021; Chen et al., 2021b; Wu et al., 2021). Therefore, the CBAM module is fused to the back of each of the three base classifiers in this paper to enhance the prediction ability of the final fusion model.

## 4. Experiment

### 4.1. Experimental Conditions

The experimental environment contains a Linux X86_64 system, an Nvidia Tesla V100 graphics card, and 16 GB memory. This experiment is based on Python version 3.7.9, TensorFlow version 2.3.0, and Keras version 2.4.3.

### 4.2. Dataset

**Figure 3:**
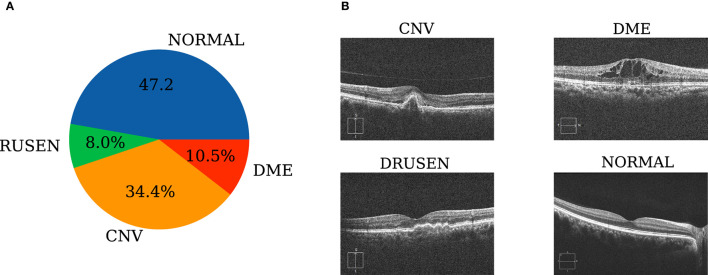
Dataset preparation. **(A)** Retinal OCT staging distribution of the samples; **(B)** representative fundus photographs of each sampling category according to their clinical diagnoses.

Internal retinal OCT images (UCSD common retinal OCT dataset, Spectralis OCT, Heidelberg Engineering, Germany) are selected from retrospective cohorts of adult patients collected by the Shiley Eye Institute of the University of California San Diego, the California Retinal Research Foundation, Medical Center Ophthalmology Associates, the Shanghai First People's Hospital, and the Beijing Tongren Eye Center between 1 July 2013 and 1 March 2017 (Kermany, 2018). The total sample size is 108,309 images, which are divided into normal, drusen, CNV, and DME images. The sample sizes of the four categories are 51,140, 8,616, 37,205 and 11,348, respectively. The data distribution is shown in Figure 3A. In addition, the internal test set sample provided by Kermany (2018) has a total of 1,000 pictures, and 250 pictures are contained in each of the four categories. The external test dataset is derived from 277 retinal OCT images provided by Beijing Chao-Yang Hospital, with CNV, DME, drusen, and normal image sample sizes of 60, 107, 27, and 83 images, respectively. Examples of each category are shown in Figure 3B.

External retinal OCT images (Cirrus HD-OCT, Carl Zeiss Meditec, USA) are selected from retrospective cohorts of adult patients collected by Beijing Chao-Yang Hospital between January 2019 and November 2021. All OCT imaging was performed as part of the patients' routine clinical care. No exclusion criteria based on age, sex, or race are included. We search local electronic medical record databases for the diagnoses of CNV, DME, drusen, and normal cases to initially assign the images. A horizontal foveal cut of the OCT scans is downloaded with a standard image format according to the manufacturer's software and instructions. Ethics Committee approvals were obtained from the Medical Ethics Review Board of Beijing Chao-Yang Hospital (2021-ke-693).

### 4.3. Evaluation Criteria

To evaluate the classification performance of the three fusion strategies employed in the proposed FN, this paper evaluates the advantages and disadvantages of the fusion strategies based on their accuracy (ACC), recall, specificity, precision, and F1 metrics.

The confusion matrix is shown in Table 2, where TP stands for the true positives, where the model predicts samples that are actually positive to be positive. FN stands for false negatives, where the model predicts samples that are actually positive to be negative. FP stands for false positives, where the model predicts samples that are truly negative as positive. TN represents true negatives, where the model predicts samples that are truly negative as negative. Therefore, the calculation formulas of the ACC, recall, specificity, precision, and F1 value metrics are as follows.

(13) $$\begin{array}{l} ACC=\frac{TP+TN}{TP+TN+FP+FN} \end{array}$$

(14) $$\begin{array}{l} Recall=\frac{TP}{TP+FN} \end{array}$$

(15) $$\begin{array}{l} Specificity=\frac{TN}{FP+TN} \end{array}$$

(16) $$\begin{array}{l} Precision=\frac{TP}{TP+FP} \end{array}$$

(17) $$\begin{array}{l} F1=\frac{2*Precision*Recall}{Precision+Recall} \end{array}$$

(18) $$\begin{array}{l} Weighted\;avg=\frac{\sum_{i=1}^{class\text{\_}num}P\text{\_}i*support\text{\_}i}{\sum_{i=1}^{class\text{\_}num}support\text{\_}i} \end{array}$$

In the formula, “ACC” represents the proportion of all correctly judged samples out of the total number of classification model samples; “Recall” represents the proportion of all outcomes in which the true value is positive and the model predicts the correct value; “Specificity” means that the true value is negative for all results, and the model predicts the correct outcomes; and “Precision” represents the proportion of model predictions among all results where the model's prediction is positive. “F1” is an indicator used to measure the accuracy of binary models in statistics. It is a harmonic average of the model accuracy rate and recall rate, and its value is between 0 and 1. The larger the value is, the better the model is. “AUC” is a performance indicator used to measure the merits and shortcomings of a model. Its value is obtained by summing the areas under the receiver operating characteristic (ROC) curve. “Weighted avg” is a weighting method that calculates the proportion of the number of samples in each category out of the total number of samples in all categories as a weight. In Formula 18, “support_i” represents the number of samples in category “i,” “P_i” represents the score value of the evaluation index of the category “i,” and “class_num” represents the number of categories.

**Table 2:** Confusion_matrix.

**Confusion_matrix**		**Predicted condition**	
		**Positive**	**Negative**
Actual condition	Positive	TP	FN
	Negative	FP	TN

### 4.4. Experimental Results

In the FN-F1-OCT FN, the weight of the base classifier needs to be artificially set to a hyperparameter. Therefore, this paper first conducts certain tests on FN-F1-OCT. In this dataset, since the F1 differences among the base classifiers on the validation sets of “Complete model,” “Limited model,” “CNV_VS_NORMAL,” “DME_VS_NORMAL,” and “Drusen_VS_NORMAL” are very small, the setting of the “n” value in the model has no great effect. Finally, the hyperparameter “n” in FN-F1-OCT is set to 0 in this paper.

**Figure 4:**
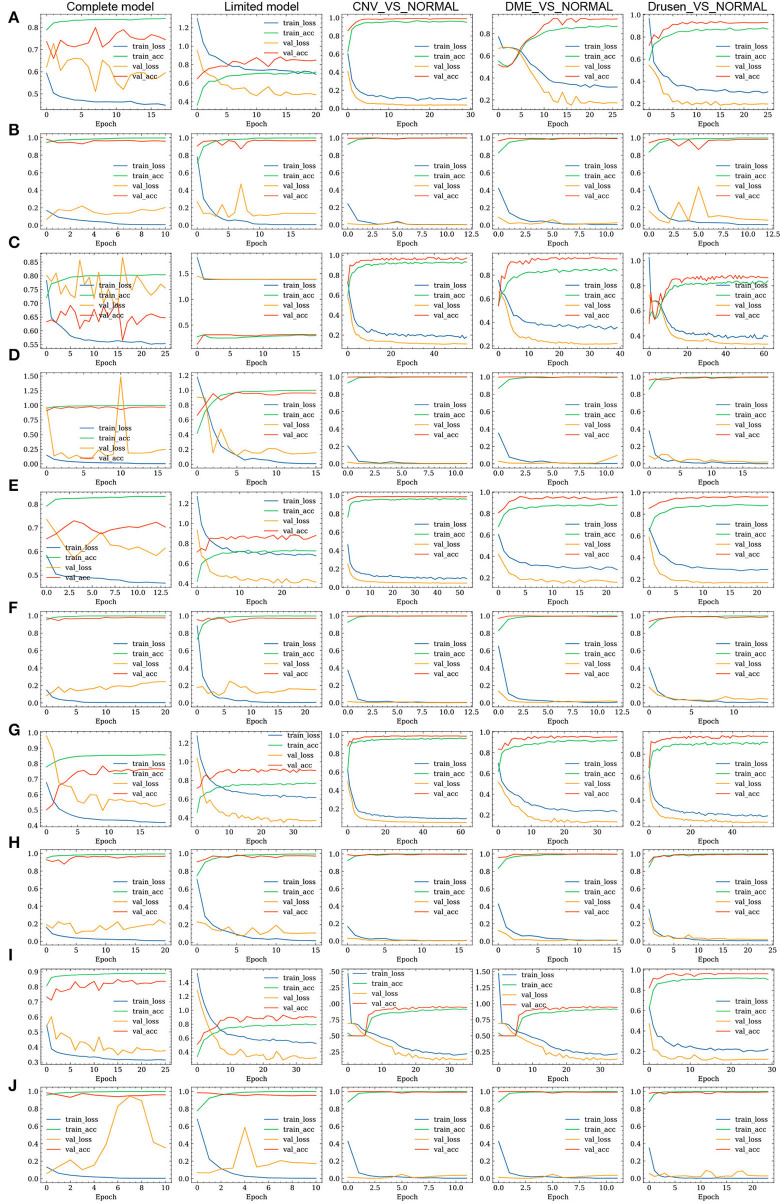
Training processes of the three fusion strategies. **(A,C,E)** represent the loss value and accuracy changes yielded on the training set and test set by the Inception, Inception-ResNet, and Xception base classifiers in FN-F1-OCT during transfer learning. **(B,D,F)** represent the fine-tuning of the models according to **(A,C,E)** in the training process, which is done to obtain the loss value and accuracy changes induced on the training set and test set. **(G,I)** represent the loss value and accuracy changes induced on the training set and test set by the FN-Weight-OCT and FN-Auto-OCT fusion strategies during transfer learning. **(H,J)** indicate that the models are fine-tuned according to **(G,I)** during the training process to obtain the loss value and accuracy changes induced on the training set and test set.

**Figure 5:**
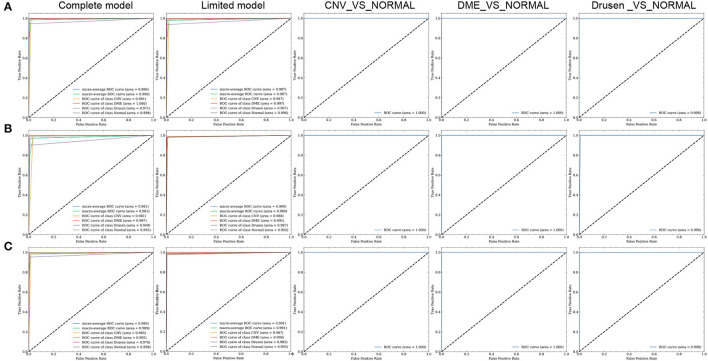
ROC curves of the three fusion strategies. **(A–C)** represent the ROC curves of FN-F1-OCT, FN-Weight-OCT, and FN-Auto-OCT, respectively.

The loss value and accuracy rate changes induced during the training processes of the FN-F1-OCT, FN-Weight-OCT, and FN-Auto-OCT FNs are shown in Figure 4. The ROC curves of FN-F1-OCT, FN-Weight-OCT, and FN-Auto-OCT are shown in Figures 5A–C, respectively. “Complete model” in Figures 4, 5 represent the use of all datasets to conduct the model training and prediction processes on the four categories (normal, drusen, CNV, and DME). “Limited model” means that 1,000 retinal OCT images are randomly selected from each category in the training set for the training and prediction of four categories: normal, drusen, CNV, and DME. “CNV_VS_NORMAL” means that 1,000 CNV and normal images are randomly selected from the training set for model training and prediction. “DME_VS_NORMAL” represents that 1,000 DME and normal random images are selected from the training set for model training and prediction. “Drusen_VS_NORMAL” represents that 1,000 drusen and normal images are randomly selected from the training set for model training and prediction. As seen in Figure 4, the loss values of the training sets of Figures 4A,C,E,G,I models vary greatly during the process of transfer learning, which is also the process of parameter adjustment in the FN-F1-OCT, FN-Weight-OCT, and FN-Auto-OCT FNs. To enhance the abilities of the fusion models to recognize retinal OCT, the loss values of Figures 4B,D,F,H,J change little during the fine-tuning process. In this process, the models undergo a fine-tuning process.

To verify the performance of the three FN-OCT implementations, a full dataset is used as a training set to compare the ACC, recall, specificity, precision, F1, and AUC values of the corresponding models. From the data in Table 3, it can be seen that the evaluation indices obtained for the drusen category are lower than those of the other categories. The main reason for this is that the drusen and CNV categories have similar reflectivity values in OCT imaging, so drusen located at the retinal pigment epithelium or below is easily considered choroid CNV; additionally, the number of drusen category images in the training dataset is too small, which makes it difficult for the models to identify the drusen category. Table 3 shows that compared with the approach of Kermany (2018), the three FN-based fusion methods proposed in this paper achieve excellent classification effects. Compared with FN-Weight-OCT and FN-Auto-OCT, FN-F1-OCT achieves better results, which is mainly due to the serious imbalance between the classes in the complete dataset, while the three base classifiers of FN-F1-OCT are trained independently. Therefore, each base classifier can generate certain compensation functions to compensate for the imbalance between categories. Compared with that of FN-Weight-OCT, FN-Auto-OCT's performance index is improved, mainly because FN-Auto-OCT's fusion mode is based on the fusion of three base classifiers after the pooling layer. Compared with FN-Weight-OCT's fusion results, FN-Auto-OCT's fusion of the pooling layer provides more weight tests in the network fusion process. Moreover, FN-Auto-OCT is based on multiple outputs, and network backpropagation mitigates the gradient disappearance problem. Therefore, FN-Auto-OCT achieves improved classification indices over those of FN-Weight-OCT.

**Table 3:** Comparison among the three developed fusion strategies in complete models.

**Complete model**		**FN-F1-OCT**	**FN-Weight-OCT**	**FN-Auto-OCT**	**Kermany**
ACC	CNV	1	0.996	0.988	0.968
	DME	1	0.996	0.996	0.948
	Drusen	0.944	0.9	0.952	0.944
	Normal	0.996	0.992	0.996	0.984
	Weighted avg	0.985	0.971	0.983	0.961
Recall	CNV	1	0.996	0.988	0.968
	DME	1	0.996	0.996	0.948
	Drusen	0.944	0.9	0.952	0.944
	Normal	0.996	0.992	0.996	0.984
	Weighted avg	0.985	0.971	0.983	0.961
Specificity	CNV	0.981	0.967	0.983	0.979
	DME	1	0.998	0.995	0.991
	Drusen	0.998	0.999	1	0.989
	Normal	1	0.999	1	0.989
	Weighted avg	0.995	0.991	0.995	0.987
Precision	CNV	0.947	0.909	0.95	0.938
	DME	1	0.992	0.984	0.971
	Drusen	0.996	0.996	1	0.967
	Normal	1	0.996	1	0.969
	Weighted avg	0.986	0.973	0.984	0.961
F1	CNV	0.973	0.951	0.969	0.953
	DME	1	0.994	0.99	0.959
	Drusen	0.969	0.946	0.975	0.955
	Normal	0.998	0.994	0.998	0.976
	Weighted avg	0.985	0.971	0.983	0.961
AUC	CNV	0.991	0.98	0.985	Not Mentioned
	DME	1	1	0.995	Not Mentioned
	Drusen	0.971	0.95	0.976	Not Mentioned
	Normal	0.998	1	0.998	Not Mentioned
	Weighted avg	0.99	0.983	0.989	Not Mentioned

For the model trained under the “Limited” setting, the overall accuracies of the three FN-OCT fusion strategies are 98, 98.4, and 98.7%. Compared with that of the model proposed by Kermany (2018), the accuracy rate is 93.4%, which is an average increase of 5 percentage points. It can be seen from the data in Table 4 that the prediction accuracy of FN-F1-OCT for drusen is still lower than that of the other categories, mainly because the FN-F1-OCT fusion strategy needs more training data than the other two fusion strategies to complete its training process, so the improvement effect on the drusen category is not good. The other fusion strategies have little differences among their evaluation indices for each category, largely solving the problems caused by data imbalance. A comparison among the various evaluation indicators is shown in Table 4. Compared with that obtained under the “Complete” setting, the evaluation indices obtained by FN-F1-OCT under the “limited” setting decline; this is mainly because the size of the training dataset decreases significantly, resulting in insufficient training. Thus, the FN-F1-OCT fusion strategy needs sufficient training data because the three base classifiers are trained separately. Compared with those of FN-F1-OCT, the evaluation indices obtained by FN-Weight-OCT and FN-Auto-OCT under the “Complete” and “Limited” settings increase to a certain extent; this is mainly because the training data in the “Limited” case are balanced data. Thus, the FN-Weight-OCT and FN-Auto-OCT fusion strategies combine the three base classifiers for training, so they do not require much training data but do require a balance between the dataset categories.

**Table 4:** Comparison among the three developed fusion strategies in limited models.

**Limited model**		**FN-F1-OCT**	**FN-Weight-OCT**	**FN-Auto-OCT**
ACC	CNV	0.996	0.988	0.98
	DME	0.996	0.984	1
	Drusen	0.936	0.98	0.976
	Normal	0.992	0.984	0.992
	Weighted avg	0.98	0.984	0.987
Recall	CNV	0.996	0.988	0.98
	DME	0.996	0.984	1
	Drusen	0.936	0.98	0.976
	Normal	0.992	0.984	0.992
	Weighted avg	0.98	0.984	0.987
Specificity	CNV	0.977	0.988	0.995
	DME	0.997	0.997	0.996
	Drusen	0.999	0.993	0.995
	Normal	1	1	0.997
	Weighted avg	0.994	0.995	0.996
Precision	CNV	0.937	0.965	0.984
	DME	0.992	0.991	0.988
	Drusen	0.995	0.98	0.984
	Normal	1	1	0.992
	Weighted avg	0.981	0.984	0.987
F1	CNV	0.966	0.976	0.982
	DME	0.994	0.987	0.994
	Drusen	0.965	0.98	0.98
	Normal	0.996	0.992	0.992
	Weighted avg	0.98	0.984	0.987
AUC	CNV	0.987	0.988	0.987
	DME	0.997	0.991	0.998
	Drusen	0.967	0.987	0.985
	Normal	0.996	0.992	0.995
	Weighted avg	0.987	0.99	0.991

To better evaluate the classification abilities of the models for the CNV, DME, drusen, and normal categories, we conduct model training and prediction for these four categories. In this paper, the ACC, recall, specificity, precision, F1, and ROC metrics in Table 5 are used for comparative analysis. According to the data in the table, FN-F1-OCT, FN-Weight-OCT, FN-Auto-OCT, and the Kermany model (2018) can achieve better classification effects for the CNV and normal categories. Compared with the Kermany model (2018), the FNs can still maintain good classification effects for the DME and normal categories, and the accuracy rate increases by 2%. Regarding the discrimination between the drusen and normal categories, the results of the three fusion strategies are consistent with Kermany's (2018) classification results.

**Table 5:** Comparison of three fusion strategies in binary classifiers.

**Binary classifiers**		**FN-F1-OCT**	**FN-Weight-OCT**	**FN-Auto-OCT**	**Kermany**
CNV _VS_	ACC	1	1	1	1
	Recall	1	1	1	1
	Specificity	1	1	1	1
	Precision	1	1	1	Not Mentioned
	F1	1	1	1	Not Mentioned
	AUC	1	1	1	1
DME _VS_ NORMAL	ACC	1	1	1	0.982
	Recall	1	1	1	0.968
	Specificity	1	1	1	0.996
	Precision	1	1	1	Not Mentioned
	F1	1	1	1	Not Mentioned
	AUC	1	1	1	0.998
Drusen _VS_ NORMAL	ACC	0.998	0.996	0.998	0.99
	Recall	0.998	0.996	0.998	0.98
	Specificity	0.998	0.996	0.998	0.992
	Precision	0.998	0.996	0.998	Not Mentioned
	F1	0.998	0.996	0.998	Not Mentioned
	AUC	0.998	0.996	0.998	0.996

To explain the application scenarios of the three fusion strategies utilized in the FNs, comparative tests are performed in this paper. Based on the number of parameters other than the weight parameters of the fixed base classifiers, the training times, the test times for 1,000 test set samples under the “Limited” setting, and the accuracies of the “Complete” and “Limit” models are compared. As shown in Table 6, the numbers of FN-F1-OCT, FN-Weight-OCT, and FN-Auto-OCT parameters do not differ much, but the training and test times of FN-F1-OCT are the longest; this is mainly because FN-F1-OCT needs to train and test the three base classifiers one by one during the training and testing processes, which in turn increases certain training costs. The training and testing times of FN-Weight-OCT and FN-Auto-OCT are not different, mainly because model training and testing are conducted by fusing three base classifiers together. Compared with FN-Weight-OCT and FN-Auto-OCT, FN-F1-OCT has certain limitations because the weight parameters of the base classifiers need to be set manually, and this process cannot be fully automated. When the data are extremely unbalanced, FN-Weight-OCT and FN-Auto-OCT perform poorly. The main reason for this is that the data imbalance causes the fusion models to become biased toward the side with more data, resulting in low data prediction accuracy. The accuracies of FN-Weight-OCT and FN-Auto-OCT increase after the data reach equilibrium. When the dataset is uneven, FN-F1-OCT can achieve a better prediction result; this is mainly because the three base classifiers of FN-F1-OCT are trained separately, and the calculation of the loss value is more accurate. However, when the number of training data decreases, the classification accuracy of the model is decreased. Therefore, the FN-F1-OCT model needs more training data to achieve excellent results.

**Table 6:** Comparison of three fusion strategies.

**Efficiency comparison**	**Parameters**	**Train time (min)**	**Test time (s)**	**“Complete” model accuracy**	**“Limited” model accuracy**
FN-F1-OCT	2,716,146	130	160	0.985	0.98
FN-Weight-OCT	2,716,149	82	124	0.971	0.984
FN-Auto-OCT	2,772,620	94	135	0.983	0.987

Therefore, the FN-F1-OCT fusion algorithm can be selected as the optimal algorithm if the training dataset contains sufficient data and time is not considered during training and testing. If the amount of training data is low and the training and testing time periods are limited, the FN-Weight-OCT fusion algorithm can be selected as the optimal algorithm. If the amount of data is low and the training and testing times are no longer considered within a certain scope, the FN-Auto-OCT fusion algorithm can be selected as the optimal algorithm.

The UCSD common retinal OCT dataset is one of the largest OCT datasets to date. It has been publicly provided by Kermany (2018) and is mainly used for retinopathy classification. To further evaluate the three fusion strategies for retinopathy extraction, this paper compares the prediction results of the three fusion strategies with the prediction results of different algorithms (Table 7). The first three rows use the prediction results obtained *via* conducting transfer learning with the classification algorithm in the ImageNet competition. Lines 4-6 use a custom algorithm or a modified version of the classification algorithm in the ImageNet contest to predict the results. From the prediction results, compared with the direct use of transfer learning without modification, the self-defined algorithm and the modified classification algorithm produce more representative retinal OCT prediction results. From lines 7–8, “Hard vote” uses the predicted class labels for majority rule voting, and “Soft vote” uses the class labels predicted based on the argmax of the sums of the predicted probabilities. It can be seen that the reason why the “Soft vote” results are consistent with the predicted labels obtained by FN-F1-OCT is that in this application scenario, the F1 value obtained by the FN-F1-OCT base classifier on the validation set is close, and the weight parameter is approximately 1/3 that of “Soft vote,” so the prediction indices are consistent. From the prediction results of lines 9–12, it can be seen that the three fusion strategies achieve greatly improved prediction abilities for retinal OCT samples thus proving the effectiveness of the proposed fusion strategies.

**Table 7:** The influences of different algorithms on the evaluation indices.

**Method**	**Acc**	**Recall**	**Specificity**
Kermany (Inception V3) (Kermany, 2018)	0.966	0.978	0.974
VGG16 (Simonyan and Zisserman, 2015)	0.939	1	0.908
ResNet50 (He et al., 2016)	0.967	0.996	0.948
Hassan (Hassan et al., 2021)	0.986	0.983	0.993
Kaymak (Kaymak and Serener, 2018)	0.971	0.996	0.984
Hwang (Hwang et al., 2019)	0.969	Not Mentioned	Not Mentioned
Hard vote	0.979	0.979	0.993
Soft vote	0.98	0.98	0.994
FN-F1-OCT	0.98	0.98	0.994
FN-Weight-OCT	0.984	0.984	0.995
FN-Auto-OCT	0.987	0.987	0.996

### 4.5. Experimental Expansion

**Figure 6:**
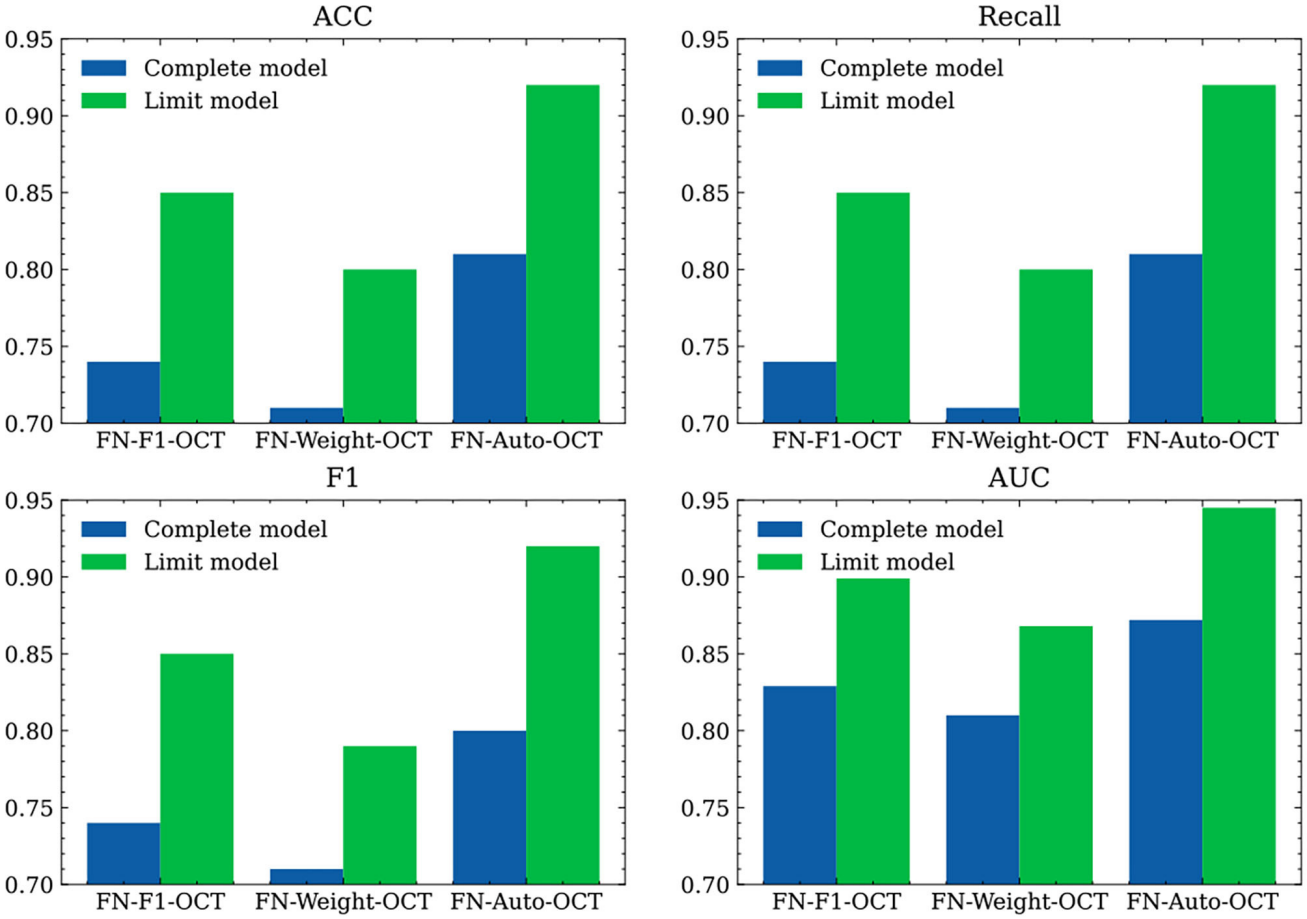
Comparison of different fusion strategies on external test datasets.

To verify the effects of the developed models on external tested data, retinal OCT images are collected from Beijing Chaoyang Hospital in this paper. We directly use the trained algorithm models to predict all image data. First, we use the preprocessing method employed on the internal test set data to process the external test set data. The evaluation indices obtained for various categories in the external test dataset are shown in Figure 6 and Table 8. In Table 8, “Weighted avg” represents the weighted average of the corresponding evaluation index, and Figure 6 shows the comparison among the weighted average results of the evaluation indices produced by the three fusion strategies. Because the network models are trained on an OCT image generated by a “Spectralis OCT” device and the test image is an OCT image generated by a “Cirrus HD” device, certain differences are observed between the definitions of the OCT images output by the two devices. Spectralis OCT equipment generally produces clearer images than Cirrus HD-OCT, which may lead to a certain decline in the evaluation indicators during model testing. As a whole, the prediction abilities of the three fusion strategies under the “Limited” setting are significantly improved compared with those obtained under the “Complete” setting; this is mainly because the training dataset used by the “Limited” models are balanced among various categories. Compared with FN-F1-OCT and FN-Weight-OCT, FN-Auto-OCT has a better generalization ability and provides better predictions on the external test sets.

**Table 8:** Comparison of different fusion strategies on external test datasets.

**External dataset**		**FN-F1-OCT**		**FN-Weight-OCT**		**FN-Auto-OCT**	
		**Complete model**	**Limited model**	**Complete model**	**Limited model**	**Complete model**	**Limited model**
ACC	CNV	0.7833	0.9167	0.8167	0.9167	0.8167	0.95
	DME	0.785	0.7663	0.757	0.7477	0.7851	0.9065
	Drusen	0.5185	0.5556	0.4445	0.2593	0.4074	0.7407
	Normal	0.7349	1	0.6747	0.9639	0.9639	0.9639
	Weighted avg	0.74	0.85	0.71	0.8	0.81	0.92
Recall	CNV	0.7833	0.9167	0.8167	0.9167	0.8167	0.95
	DME	0.785	0.7663	0.757	0.7477	0.7851	0.9065
	Drusen	0.5185	0.5556	0.4445	0.2593	0.4074	0.7407
	Normal	0.7349	1	0.6747	0.9639	0.9639	0.9639
	Weighted avg	0.74	0.85	0.71	0.8	0.81	0.92
F1	CNV	0.7768	0.8397	0.7968	0.7746	0.7778	0.9120
	DME	0.7636	0.8677	0.7232	0.8377	0.8442	0.9327
	Drusen	0.5714	0.6250	0.5455	0.3415	0.4889	0.7145
	Normal	0.7439	0.8925	0.6871	0.8889	0.8695	0.9697
	Weighted avg	0.74	0.85	0.71	0.79	0.8	0.92
AUC	CNV	0.859	0.921	0.876	0.896	0.869	0.957
	DME	0.807	0.883	0.773	0.862	0.869	0.942
	Drusen	0.743	0.766	0.712	0.616	0.69	0.852
	Normal	0.816	0.948	0.775	0.938	0.928	0.977
	Weighted avg	0.829	0.899	0.81	0.868	0.872	0.945

### 4.6. FN Visualization

**Figure 7:**
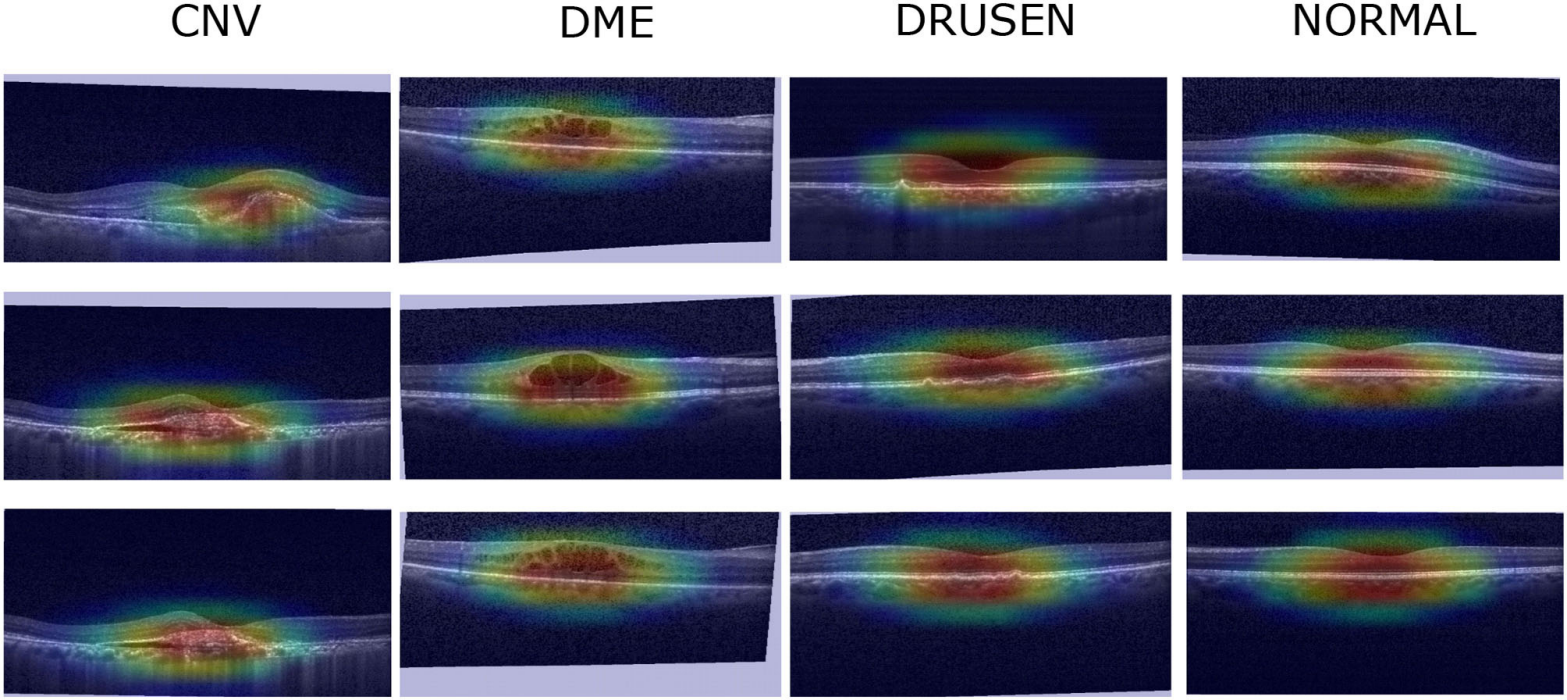
Localization map visualization.

To verify the effectiveness of the FNs proposed in this paper, the three FNs are used for clinical verification in a localization map task. First, the three images with the highest prediction probabilities in each category are selected as visualization images, and then the Grad-CAM (Selvaraju et al., 2017) algorithm is used visualize a localization map, which is subsequently checked by professional doctors. The localization map of the FN-Auto-OCT classification results obtained after the final evaluation index of Xception goes through a CBAM (Figure 7). After the image is checked by professional clinicians, the visual part of the image can show the locations at which the model focus is similar to human experience. The OCT of CNV is characterized by interlayer effusion, lipid exudation, and irregularly raised retinal pigment epithelium (RPE) with a widened fusiform band due to broken choroidal capillaries. DME manifests in OCT as retinal cystic changes at the macular fovea, decreased signal reflection in the lumen, and swollen retinal inner surfaces. Enhanced reflectance of the choroid and RPE can be found in the OCT of drusen, which is accompanied by RPE focal protrusions. In the OCT of the normal group, the retina is clearly stratified and well structured, thus verifying the effectiveness of the examined FN.

## 5. Conclusion

In this paper, an FN-based retinal OCT algorithm for retinal detection is proposed. The experimental results show that this paper explores the fusion modes of FNs in three ways, which can provide the base classifiers with strong retinal OCT detection abilities. The results of comparisons with related approaches confirm the accuracy of the developed algorithm. In the future, we plan to use other local HD image databases to check the robustness of the proposed algorithm, and we will apply the three fusion strategies in other application scenarios to verify the advantages of this algorithm.

## Data Availability Statement

The internal raw data are provided by (Kermany, 2018) and are directly available for download (https://data.mendeley.com/datasets/rscbjbr9sj/3). The internal test set is not publicly accessible due to the privacy concerns associated with clinical data, but the internal test set can be supported by data from the corresponding authors upon reasonable request. All deep learning methods are implemented by using TensorFlow (https://tensorflow.google.cn/). The custom-written scripts for this study are available upon reasonable request.

## Author Contributions

ZA, XH, and JF: conceptualization. ZA and XH: methodology. ZA and YL: writing of the original draft. ZA, HW, and FZ: review and editing of the writing. YL, YT, and FZ: project administration. XH, JF, and HW: data collection. FZ, XH, HW, and YL: funding acquisition. All authors read and agreed to the published version of the manuscript.

## Funding

This work was supported in part by a grant from the National Natural Science Foundation of China to FZ (81902861), in part by a grant from the National Natural Science Foundation of China to XH (32000485), in part by a grant from the National Natural Science Foundation of China to HW (62006161), and in part by Sinopharm Genomics Technology Co., Ltd. The funders were not involved with the study design, collection, analysis, data interpretation, the writing of this article or the decision to submit it for publication.

## Conflict of Interest

ZA and YL were employed by Sinopharm Genomics Technology Co., Ltd. The remaining authors declare that the research was conducted in the absence of any commercial or financial relationships that could be construed as potential conflicts of interest.

## Publisher's Note

All claims expressed in this article are solely those of the authors and do not necessarily represent those of their affiliated organizations, or those of the publisher, the editors and the reviewers. Any product that may be evaluated in this article, or claim that may be made by its manufacturer, is not guaranteed or endorsed by the publisher.
